# A Multidisciplinary Approach to the Diagnosis and Management of a Mammary Myofibroblastoma in a Male with a History of Diffuse Large B-Cell Lymphoma: A Case Report

**DOI:** 10.3390/hematolrep18020023

**Published:** 2026-03-17

**Authors:** Carmen Montes Fernández, Norma C. Gutiérrez, Elena Alejo Alonso, Susana Gallego García, Luis Gonzaga Díaz-González, José Luis Revilla Hernández, María Ángeles Hernández García, Idalia González Morais, Miguel Ángel Cruz Sánchez, José María Sayagués, Luis Miguel Chinchilla-Tábora

**Affiliations:** 1 Department of Hematology, Virgen de la Concha Hospital, 49022 Zamora, Spain; mcmonte@saludcastillayleon.es; 2 Department of Hematology, University Hospital of Salamanca, 37007 Salamanca, Spain; ncgutierrez@saludcastillayleon.es (N.C.G.); mariangeles@usal.es (M.Á.H.G.); 3 Molecular Cytogenetics Unit of Salamanca, Cancer Research Center (CIC), University Hospital of Salamanca, 37007 Salamanca, Spain; 4 Institute for Biomedical Research of Salamanca (IBSAL), 37007 Salamanca, Spain; lgdiaz@saludcastillayleon.es (L.G.D.-G.); ppmari@usal.es (J.M.S.); 5 Department of Radiology, Virgen de la Concha Hospital, 49022 Zamora, Spain; sgallegoga@saludcastillayleon.es; 6 Department of Nuclear Medicine, University Hospital of Salamanca, 37007 Salamanca, Spain; 7 Department of Surgery, Virgen de la Concha Hospital, 49022 Zamora, Spain; jlrevilla@saludcastillayleon.es; 8 Department of Pathology, University Hospital of Salamanca, 37007 Salamanca, Spain; igonzalezmor@saludcastillayleon.es (I.G.M.); miguelacruz@saludcastillayleon.es (M.Á.C.S.); 9 Department of Pathology, Virgen de la Concha Hospital, 49022 Zamora, Spain

**Keywords:** myofibroblastoma, diffuse large B-cell lymphoma, mammary myofibroblastoma, multidisciplinary team, immunohistochemistry, fluorescence in situ hybridization

## Abstract

**Background and Clinical Significance**: Diffuse Large B-Cell Lymphoma (DLBCL) is a morphologically and molecularly heterogeneous lymphoproliferative disorder that originates from a clonal B-cell ancestor. Patients usually present with rapidly enlarging lymph nodes or mass(es) at single or multiple sites. Generally, 18F-Fluorodeoxyglucose (18F-FDG) positron emission tomography with computed tomography (PET-CT) is performed post-treatment to evaluate remission status, especially in radiologically residual tumors. Myofibroblastoma (MFB) is a benign mesenchymal tumor of the mammary stroma composed of fibroblasts and myofibroblasts. These entities do not often present concurrently. **Case presentation**: The patient was an 80-year-old man with a history of stage IV-BS Diffuse Large B-Cell Lymphoma (DLBCL) with a high-risk International Prognostic Index (IPI). The patient underwent treatment with a six-cycle R-CHOP regimen. Immediately after the last cycle, an 18F-Fluorodeoxyglucose (18F-FDG) positron emission tomography with computed tomography (PET-CT) scan revealed a nodular solid lesion with a faintly increased metabolic standardized uptake value (SUVmax) of 3 in the upper outer quadrant of his left breast. A biopsy of the breast lesion was performed, and it revealed a benign mesenchymal tumor, specifically a Myofibroblastoma. The patient has not presented any symptoms or complications since surgery (12 months) and remains in complete remission (CR). **Conclusions**: Given the potential diagnostic pitfalls and therapeutic implications of residual tumors in the context of DLBCL, a conscientious evaluation by a multidisciplinary team (MDT) is highly recommended.

## 1. Introduction

Myofibroblastoma (MFB) is a benign tumor of the mammary stroma composed of fibroblasts and myofibroblasts, usually presenting as a slow-growing, painless, non-tender mass [1]. Females and males are equally affected by MFB, and most MFB cases are seen in elderly men and postmenopausal women [2].

The mesenchymal lesions encountered in breast specimens may originate from the mammary parenchyma and its associated skin, subcutaneous tissue, deep soft tissue, or even any of its mesenchymal elements (vascular, fibroblastic/myofibroblastic, adipocytic, peripheral nerve, and smooth muscle) [3].

The essential and desirable diagnostic criteria for MFB proposed by the WHO in its 5th edition regarding breast tumor classification are as follows: essential criteria: well-circumscribed margins, a mesenchymal tumor without epimyoepithelial components, none or only mild nuclear atypia or pleomorphism, a low mitotic count, and short interlacing fascicles; desirable criteria: positive immunohistochemistry for Desmin, CD34, estrogen receptors (ERs), progesterone receptors (PRs), and androgen receptors (ARs) and FISH: 13q14 deletion [4,5].

Diffuse Large B-Cell Lymphoma (DLBCL) not otherwise specified (NOS) is a lymphoma consisting of medium-to-large sized B-cells with a diffuse growth pattern and represents 30% of adult lymphoma cases worldwide [6].

Synchronous primary malignancies of the breast, colon, and prostate as well as other solid cancers involving DLBCL have been reported in the reviewed literature [7,8]. The influence of chemotherapy and radiation therapy on second primary malignancies (SPMs) in patients with DLBCL is well known [9].

The treatment for multiple tumors involving lymphoma requires individualized planning based on the stage and pathological type of each tumor, considering the patient’s overall condition. Once the pathological diagnosis is known, each tumor should be staged independently. Given the potential for confusion between metastatic or relapsed cancer, multidisciplinary team (MDT) discussions are highly recommended for challenging cases [8].

We present a case of a male patient with a mammary MFB, who was previously diagnosed and treated for Diffuse Large B-Cell Lymphoma in another medical institution, emphasizing the relevance of the MDT approach for correct diagnosis and treatment.

## 2. Case Presentation

**Figure 1:**
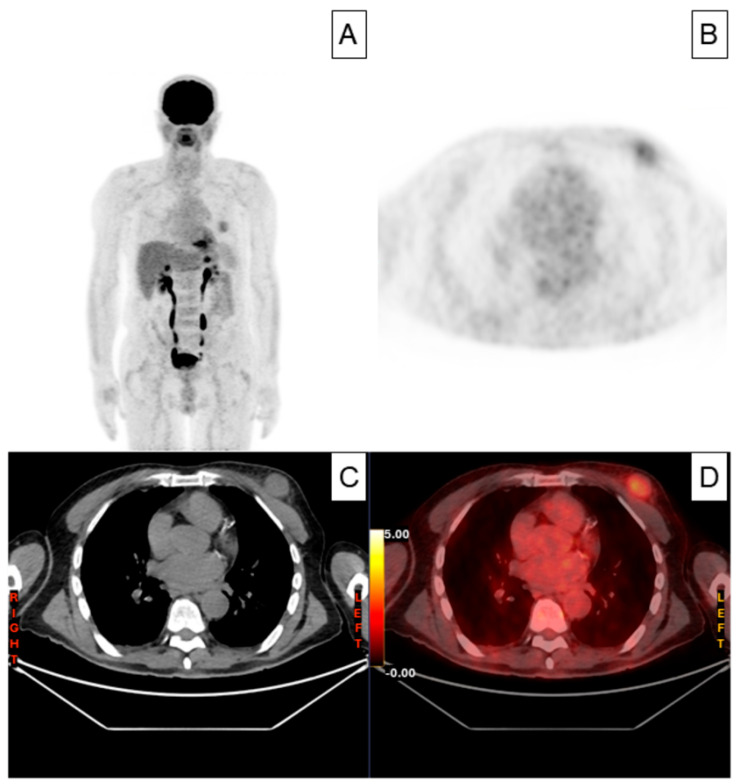
(**A**–**D**) 18F-Fluorodeoxyglucose (18F-FDG) positron emission tomography with computed tomography (PET-CT) showing a nodular solid lesion with a faintly increased metabolic standardized uptake value (SUVmax) of 3 located in the upper outer quadrant of his left breast.

The patient was an 80-year-old man with a history of stage IV-BS Diffuse Large B-Cell Lymphoma (DLBCL) and a high-risk International Prognostic Index (IPI) who was initially diagnosed and treated in another medical center. The patient underwent six doses of systemic treatment with the R-CHOP regimen. Once the treatment had finished, no adenomegaly or visceromegaly were found on physical examination. The patient had not reported fever or night sweats, and he had gained weight during the last months (no disease symptoms). The patient underwent 18F-Fluorodeoxyglucose (18F-FDG) positron emission tomography with computed tomography (PET-CT) scanning immediately after the completion of the six-cycle R-CHOP protocol, which evidenced a partial response with decreased uptake and size of the lymph nodes; however, paradoxically, a nodular solid lesion with a faintly increased metabolic standardized uptake value (SUVmax) of 3 was detected in the upper outer quadrant of his left breast (Figure 1A–D).

**Figure 2:**
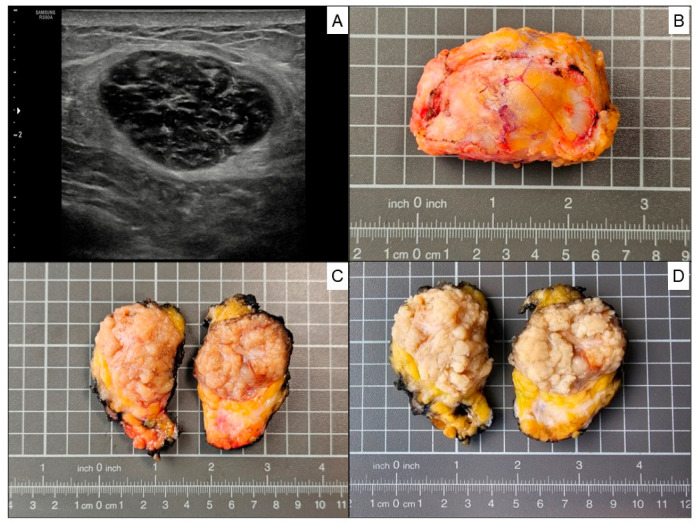
(**A**) Breast ultrasonography showing a well-circumscribed hypoechoic nodule (largest diameter: 3.7 cm). (**B**,**C**) The gross pathology of a surgical specimen showing a well-circumscribed, unencapsulated tumor, measuring 4.5 × 3.4 × 1.8 cm, with a nodular yellow-to-whitish/gray cut surface before fixation and (**D**) a light tan color after formaldehyde fixation.

A breast ultrasonographic examination was conducted showing a well-circumscribed hypoechoic breast nodule and its largest diameter was 3.7 cm, located in the upper outer quadrant of the left breast (Figure 2A). A 14G ultrasound-guided needle biopsy was performed, followed by a biopsy site marker procedure for the precise marking of the biopsy site (Tumark^®^ Eye, SOMATEX Medical Technologies GmbH-HOLOGIC, Kaiserin-Augusta-Allee 112/113, 10553 Berlin, Deutschland). The radiological image indicated a BI-RADS 3-category lesion (probably benign). The pathology report indicated “spindle cell-patterned mesenchymal proliferation suggestive of myofibroblastoma”. An explanatory note was added to the pathology report, urging that the complete tumor study would be necessary to reach a definitive diagnosis. After analyzing the case in the multidisciplinary team, it was decided to perform surgery to completely remove the breast tumor. The surgical specimen showed a well-circumscribed, unencapsulated tumor with a nodular yellow to whitish/gray cut surface before formaldehyde fixation (Figure 2B,C) and a light tan color after formaldehyde fixation (Figure 2D), measuring 4.5 × 3.4 × 1.8 cm.

**Figure 3:**
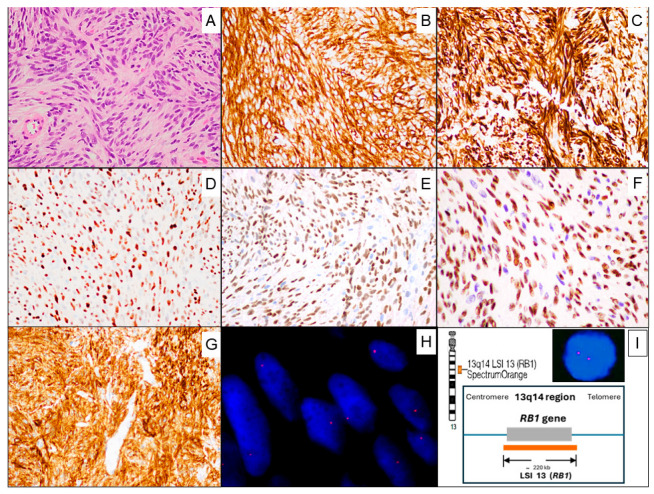
(**A**) The tumor consists of monotonous cells, displaying a pale-to-eosinophilic cytoplasm, arranged randomly or in short intersecting fascicles. A vaguely storiform pattern is discernible in some areas. The nuclei are round to oval. Hematoxylin and eosin (H&E) staining. (**B**) CD34 immunoreactivity. (**C**) Desmin immunoreactivity. (**D**) Estrogen receptor immunoreactivity. (**E**) Progesterone receptor immunoreactivity. (**F**) Androgen receptor immunoreactivity. (**G**) CD10 immunoreactivity. (**H**) FISH analysis of Myofibroblastoma using a Vysis LSI 13 (RB1) 13q14 SpectrumOrange Probe showing the loss of one signal of RB1 gene (deletion at 13q14 region) in tumor cell nuclei. (**I**) Ideogram of chromosome 13 showing the mapping position of the RB1 gene (upper left side). Diagram showing the FISH probes for the RB1 gene (center). Image of LSI 13 (RB1) 13q14 Probe hybridized to a normal nucleus (of a non-tumoral cell) showing a two-orange signal pattern (upper right side).

A microscopic examination revealed mesenchymal tumor proliferation with well-circumscribed, unencapsulated borders. It consisted of spindle-to-oval-shaped, monotonous cells with little cytological atypia, displaying a pale-to-eosinophilic cytoplasm, arranged randomly or in short intersecting fascicles with fine bundles of hyalinized collagen between the cells. A vaguely storiform pattern was discernible in some areas. The nuclei were round to oval; some of them displayed small, barely evident nucleoli and pseudoinclusions. Mitoses were very rare (1 in 20 high-power fields), without atypical mitotic figures. No entrapment of mammary ducts or lobules was observed. Necrosis was absent. The stroma was fibrous and contained thin-walled capillaries, without a defined organizational pattern (Figure 3A). In addition, a small number of mature lymphocytes that showed leukocyte common antigen (LCA/CD45) immunoreactivity and few mast cells that showed CD117 positivity were observed among the tumor cells.

Immunohistochemistry revealed that tumor cells were positive for CD34 (Figure 3B), Desmin (Figure 3C), estrogen receptors (ERs) (Figure 3D), progesterone receptors (PRs) (Figure 3E), androgen receptors (ARs) (Figure 3F), CD10 (Figure 3G), and focally Muscle-Specific Actin (MSA). By contrast, the tumor cells were negative for Cytokeratins (AE1–AE3), S100, STAT6, ALK, β-catenin, CD117, CD31, leukocyte common antigen (CD45), and B-cell markers (CD20, CD79a, and PAX5).

Fluorescence in situ hybridization (FISH) was performed and analyzed at our Molecular Cytogenetics Unit to determine the status of 13q14 (RB1). Two-micrometer sections of formalin-fixed paraffin-embedded (FFPE) tumor tissue were used, and then the Vysis LSI 13 (RB1) 13q14 SpectrumOrange Probe was utilized. The FISH analysis showed a loss of one signal of RB1 (deletion at the 13q14 region) in 84% of the tumor cell nuclei (Figure 3H), confirming the diagnosis of MFB.

During the follow-up, the patient has not presented any symptoms or complications since surgery (12 months) and remains in complete remission (CR) of lymphoma.

## 3. Discussion

In recent decades, the introduction of effective therapies like R-CHOP has had a significant impact on the cure rates of Diffuse Large B-Cell Lymphoma (DLBCL), leading to an increase in number of long-term survivors [10]. Nevertheless, some of these long-term survivors develop secondary primary malignancies (SPMs) or benign tumors, as well as complications directly or indirectly related to the treatment modality [11], and the age at diagnosis [9,12], the time since the DLBCL diagnosis, and the stage of the disease at diagnosis [11] are important factors affecting survival.

In our case, a breast Myofibroblastoma appeared at the time when the primary DLBCL was expected to be in remission. Moreover, it was identified as the primary new finding obtained from the definitive restaging PET-CT performed immediately after the completion of six cycles of R CHOP.

Given the early timing of therapy completion, this case does not exhibit the classical pattern of a late, therapy-related second primary malignancy. Instead, we use the concept of “second tumors in DLBCL survivors” to illustrate how benign lesions can mimic relapse or progression on imaging, thereby creating significant diagnostic uncertainty.

In our case, the relatively low SUVmax (3) and the BI-RADS 3 assessment argued against aggressive DLBCL relapses, but considering the patient’s age, recent treatment, and oncologic history, a tissue diagnosis remained essential.

The wide spectrum of secondary tumors in patients with a history of DLBCL requires a multidisciplinary team (MDT) approach that includes at least one hematologist, one radiologist, one nuclear medicine specialist, one surgeon, and one pathologist [10,13].

Various cases of DLBCL combined with one or two different primary tumors have been reported in recent decades [8].

Extramammary MFB has been described, and it is often referred to as “mammary-type” Myofibroblastoma (MTMF) when occurring at other sites [14]. In this way, MTMF has been reported at different anatomic locations including the inguinal/groin region [15], chest wall [16], axilla [17], lower and upper extremities [18], intra-abdominal/retroperitoneal region [19], and liver [20,21]. Therefore, it is likely that any of these locations could be misdiagnosed as the metastasis or relapse of DLBCL in follow-up radiological tests.

Immunohistochemistry is useful for distinguishing MFB from other tumors included in the differential diagnosis, such as metaplastic spindle cell carcinoma (in cases located in the breast), nodular fasciitis, fibromatosis, pseudoangiomatous stromal hyperplasia (PASH), solitary fibrous tumor, inflammatory myofibroblastic tumor, and spindle cell lipoma, among others [3,22]. Therefore, the immunohistochemical panel used should include the following antibodies: Cytokeratin (AE1-AE3), STAT6, ALK, β-catenin, EMA, S100, and others [23,24,25]. Our case also showed immunoreactivity for CD10 in concordance with the reported cases by Gaetano Magro et al. [26].

Monoallelic 13q14 deletion and the RB1 loss identified by immunohistochemistry are not specific chromosomal alterations of MFB. Other 13q14-deleted mesenchymal tumors, such as spindle cell/pleomorphic lipomas (SCLs) and cellular angiofibromas (CAFs), were considered and excluded in the differential diagnosis based on histology and immunoprofile [27]. Monoallelic 13q14 deletion and the RB1 loss have also been identified in a significant subset of cases of atypical spindle/pleomorphic lipomatous tumors (ASPLTs) [28]. Based on the clinicopathological context of our case, it is of interest to know that 13q14 deletion is a recurrent event in some hematologic neoplasms such as chronic lymphocytic leukemia/small lymphocytic lymphoma (CLL/SLL) [29,30] and in a subset of DLBCL [31], but in our patient, DLBCL did not show 13q14 deletion.

In our case, the metastasis or relapse of DLBCL was ruled out based on the spindle cell morphology of the tumor cells, the benign histological pattern, and the lack of lymphoid immunohistochemical markers (leukocyte common antigen (CD45) and B-cell markers (CD20, CD79a, and PAX5)).

It is worth nothing that the most frequent primary lymphoma of the breast is in fact DLBCL [32]. However, any type of lymphoma can occur as a primary breast lymphoma (PBL). A PBL is a rare condition, accounting for less than 0.5% of all breast malignancies [33].

In contrast to the extensively documented incidence of second malignant neoplasms in patients with lymphoma [9,34,35,36], there is not enough evidence addressing the potential association between lymphomas and the subsequent development of benign tumors. Sometimes, these tumors can be misdiagnosed as lymphoma relapses or disease progression on imaging. In this context, the 18F-FDG PET-CT can help to clarify the nature of second tumors. To the best of our knowledge, this is the first case report in the medical literature of a mammary MFB arising in a male in the context of DLBCL. The constellation of histological features supported by the monoallelic loss of 13q14 region detected by FISH and the RB1 loss detected by immunohistochemistry, in the appropriate clinical context, confirms the diagnosis of MFB, thus avoiding its overtreatment.

## 4. Conclusions

Since there are no established guidelines on how to proceed with patients with MFB in the context of DLBCL, it is necessary to develop standards for the management of these cases. Our study is based on a single case, and we recognize the inherent limitation of generalizability from a single case. Based on our experience with this case, we suggest the following diagnostic and therapeutic workflow: (1) First, the case should be evaluated by a multidisciplinary team (MDT) that must include at least one hematologist, one radiologist, one nuclear medicine specialist, one surgeon, and one pathologist. (2) The correct interpretation of imaging (especially 18F-Fluorodeoxyglucose (18F-FDG) positron emission tomography with computed tomography (PET-CT)) can help to distinguish lymphoma relapses from a second benign tumor. If doubts persist, (3) a biopsy with adequate tissue for histology and immunohistochemical and molecular techniques should be performed. The pathology report confirms the tumor cells’ nature (benign or malignant; lymphoid, epithelial, or mesenchymal lineage). If pathology confirms Myofibroblastoma (MFB) and there are no clinical or surgical contraindications, (4) the complete excision of the tumor(s) with clear margins is the treatment of choice. Finally, (5) following the definitive treatment, the standardized clinical and radiological follow-up must be carried out to detect lymphoma relapses, MFB local recurrence, or the appearance of new masses.

Malignant and benign tumors can mimic a DLBCL relapse on PET-CT; therefore, an MDT evaluation including a biopsy is critical to avoid unnecessary systemic therapy or overtreatment.

Proper management in cases like ours by an MDT is critical to decide the best therapeutic options for the patients. The correct integration of clinical data with radiological and histopathological findings may prevent misdiagnosis and inappropriate treatment in daily practice.

Prospective series or multi-center collections of similar benign “second tumors” in lymphoma survivors would help quantify their true incidence and refine management algorithms.

## Data Availability

The original contributions presented in this study are included in the article. For any further inquiries, please contact the corresponding author.

## Abbreviations

The following abbreviations are used in this manuscript:

MFB	Myofibroblastoma
MTMF	“Mammary-type” Myofibroblastoma
DLBCL	Diffuse Large B-Cell Lymphoma
NOS	Not Otherwise Specified
SPMs	Second Primary Malignancies
PBL	Primary Breast Lymphoma
MDT	Multidisciplinary Team
PET-CT	Positron Emission Tomography with Computed Tomography
18F-FDG	18F-Fluorodeoxyglucose
SUV	Standardized Uptake Value
FFPE	Formalin-Fixed Paraffin-Embedded
FISH	Fluorescence In Situ Hybridization
ER	Estrogen Receptor
PR	Progesterone Receptor
AR	Androgen Receptor
